# Correction

**DOI:** 10.1111/pbi.14361

**Published:** 2024-05-24

Correction to ‘CRISPR/Cas9‐mediated seamless gene replacement in protoplasts expands the resistance spectrum to TMV‐U1 strain in regenerated *Nicotiana tabacum*’

Li, Y., Huang, C., Liu, Y., Zeng, J., Yu, H., Tong, Z., Yuan, X., *et al*. (2023) CRISPR/Cas9‐mediated seamless gene replacement in protoplasts expands the resistance spectrum to TMV‐U1 strain in regenerated *Nicotiana tabacum*. *Plant Biotechnol. J*. **21**, 2641–2653.

In the above article, the authors would like to update Figure 3e. The images should show two different leaves, however, the published version mistakenly contains the same picture reproduced twice. The correct figure is shown below.

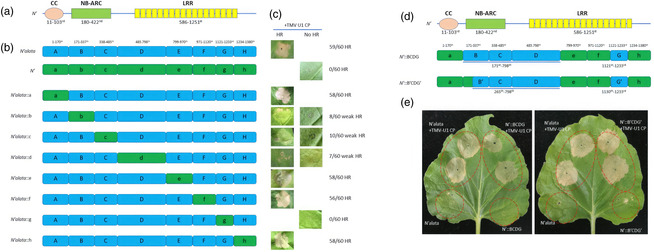

**Figure 3** Verification of the *N′ alata* gene resistance‐determining regions. (a) Schematic diagram of the *N′* gene domain structure. CC represents the coiled‐coil domain. NB‐ARC represents the nucleotide‐binding adaptor shared by the APAF‐1, R proteins and the CED‐4 domain. LRR represents the leucine‐rich repeat domain. The amino acid distributions are labelled below each domain. (b) Schematic diagram of *N′ alata*, *N′* and recombinant *N′ alata* with fragments swapped from the *N′* gene. The *N′ alata* and *N′* genes were divided into eight regions to facilitate construction of different domains. The amino acid ranges are labelled for each domain. *N′ alata*, *N′* and recombinant *N′ alata* genes were inserted between the 35S promoter and octopine synthase terminator. (c) Transient co‐expression of the *N′ alata*, *N′* and recombinant *N′ alata* genes along with the TMV‐U1 CP gene in *N. benthamiana* leaves. Two days after agroinfiltration, the *N. benthamiana* leaves exhibited hypersensitive response (HR) symptoms (left) or no HR symptoms (right). The HR implies that the recombinant gene is resistant to TMV‐U1, whereas the absence of HR indicates susceptibility to TMV‐U1. The proportions of infiltration sites showing the HR for each construct are shown based on counting 60 infiltration sites per construct in three independent experiments. Under most circumstances, the *N′ alata::b*, *N′ alata::c* and *N′ alata::d* genes failed to induce HR symptoms, but they occasionally induced weak HR. Other constructs induced only one dominant symptom after infiltration. (d) Schematic diagram of the *N′* gene with fragments swapped from the *N′ alata* gene. The BCD and G regions were truncated into the B'CD and G' regions, respectively, to more precisely locate the resistance‐determining regions. (e) Transient co‐expression of recombinant *N′* genes with TMV‐U1 CP compared with only the recombinant *N′* genes in *N. benthamiana* leaves. The Infiltrated areas of each structure are indicated with dotted red circles. Transient expression of recombinant *N′* genes without the TMV‐U1 CP was used as controls to eliminate spontaneous HR responses to infiltration. The *N′* gene is shown in green and the *N′ alata* gene is in cyan. All infiltration assays were repeated three times and a total of 60 infiltrations were conducted for each structure.

We apologize for these errors.

